# Disruption of the *mutator* complex triggers a low penetrance larval arrest phenotype

**Published:** 2020

**Authors:** Alicia Kathryn Rogers, Carolyn Marie Phillips

**Affiliations:** 1 Department of Biological Sciences, University of Southern California, Los Angeles, California, United States of America

## Description

*Mutator* foci are perinuclear granules in the germline of *Caenorhabditis elegans* that are required for the amplification of 22G-small interfering RNAs (siRNAs) (Phillips et al., 2012). These *mutator*-dependent siRNAs act downstream of primary endogenous and exogenous siRNA pathways and are necessary for robust and heritable silencing (Pak et al., 2007; Sijen et al., 2007; Gu et al., 2009; Gent et al., 2010; Vasale et al., 2010; Phillips et al., 2012). There are numerous factors that have been identified that localize to *Mutator* foci and are required for *mutator*-dependent siRNA biogenesis. These *mutator*-class proteins include the core component of *Mutator* foci MUT-16, the nucleotidyl transferase MUT-2, the 3’-5’ exonuclease MUT-7, the DEAD-box RNA helicases MUT-14 and SMUT-1, the Zc3h12a-like ribonucleases RDE-8, NYN-1, and NYN-2, and two proteins of unknown function, MUT-15 and RDE-2 (Ketting et al., 1999; Tijsterman et al., 2002; Vastenhouw et al., 2003; Chen et al., 2005; Tops et al., 2005; Phillips et al., 2012; Phillips et al., 2014; Tsai et al., 2015). Additionally, the RNA-dependent RNA polymerase RRF-1 localizes to *Mutator* foci but is redundant with EGO-1 for *mutator*-dependent siRNA biogenesis (Phillips et al., 2012; Gu et al., 2009). It was previously shown that mutations in *mutator*-class genes are sterile at elevated temperature (Ketting et al., 1999; Zhang et al., 2011; Rogers and Phillips, 2020). Recently, we performed a brood size assay using wild-type and *mut-16* hermaphrodites cultured at 20°C. We found that compared to wild-type animals, *mut-16* mutant animals lay fewer eggs (56% fewer eggs laid compared to wild-type animals), and of those eggs, fewer *mut-16* mutant eggs hatch (81% of *mut-16* mutant eggs hatch compared to wild-type, where 100% of the eggs hatch) (Rogers and Phillips, 2020). Furthermore, 100% of wild-type larvae successfully mature to adulthood, whereas only 85% of *mut-16* mutant larvae mature to adulthood (Rogers and Phillips, 2020). The reduced hatching rates and larval arrest of *mut-16* mutant animals had not been previously reported.

**Figure 1:**
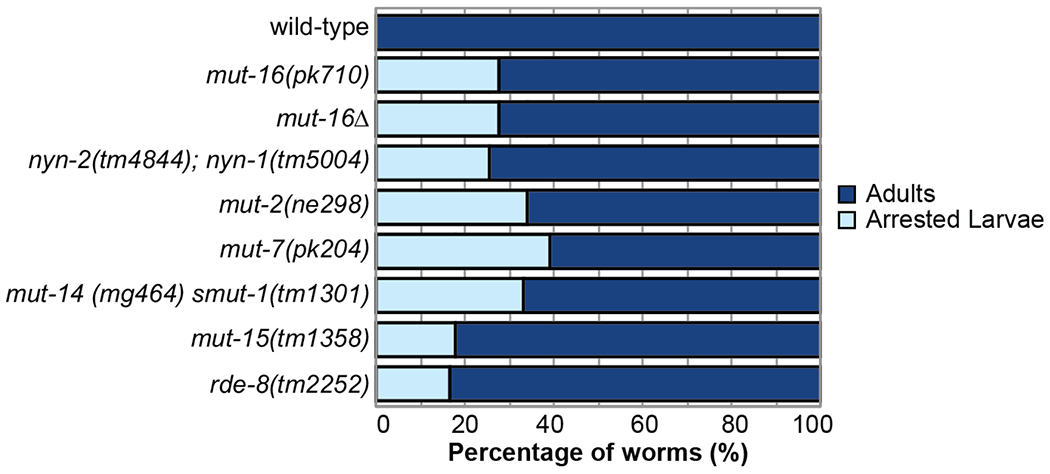
Larval arrest is a shared phenotype of *mutator* mutants. Bar graph shows percentage of synchronized L1 larvae for each *mutator* mutant strain that reach adulthood (dark blue) or arrest at any larval stage (light blue) when cultured at 20°C. For each strain, n=1,000 animals.

Because one phenotype of mutants of the *mutator*-class genes is hopping of transposable elements, and thus they can exhibit spontaneous mutations (Ketting et al., 1999), in this work we first sought to test whether the larval arrest phenotype is found in other *mut-16* mutant alleles and not due to a background mutation in the *mut-16(pk710)* strain. We performed a larval arrest assay in which we counted the total number of individuals that mature to adulthood or arrest as larvae for wild-type (N2) and *mut-16* mutants. We used two *mut-16* alleles, *mut-16(pk710)*, the same allele as the original assay which carries an early stop codon, and *mut-16(cmp185)* (referred to here as *mut-16Δ*), an in-frame 560 amino acid deletion (Uebel et al., 2018). One thousand L1 stage animals of each strain were plated at 20°C. After seventy-two hours, the developmental stage of the individuals was assessed. We found that 28% of both *mut-16Δ* and *mut-16(pk710)* mutant individuals arrested as larvae compared to 0% of wild-type individuals (Figure 1).

To determine whether larval arrest is a common phenotype amongst other *mutator* mutants, we counted the larval arrest rate for six additional *mutator*-class mutants. We observed that a portion of the L1 stage animals from each *mutator*-class mutant arrested at either the L1 or L2 stage – *nyn-1(tm5004); nyn-2(tm4844)* mutants (26% arrest), *mut-2(ne298)* mutants (34% arrest), *mut-7(pk204)* mutants (39% arrest), *mut-14(mg464) smut-1(tm1301)* mutants (33% arrest), *mut-15(tm1358)* mutants (18% arrest), and *rde-8(tm2252)* mutants (16% arrest) (Figure 1). These data indicate that larval arrest is a low penetrance phenotype found in between 16% and 39% of mutant individuals, and that *mutator*-class proteins, such as MUT-16, play an important role in ensuring the development of *C. elegans*.

While elevated temperature triggers sterility in *mutator*-class mutants, here we show that *mutator*-class mutants also exhibit a larval arrest phenotype at permissive temperature. Larval arrest can occur in *C. elegans* for many reasons, including but not limited to, stressful conditions such as starvation – which could occur due to a lack of food (Johnson et al., 1984), the inability to consume food or perform pharyngeal pumping (Fay et al., 2003; Furuya et al., 2005; Mango, 2007), or the inability to absorb nutrients in the gut (Thieringer et al., 2003) – or mis-regulation of cell cycle components (Boxem et al., 1999; van den Heuvel, 2005), proteasome components (Takahasi et al., 2002), or other pathways that affect development. The individuals assayed in our experiments were not grown under stressful conditions or on densely populated plates. Thus, the underlying cause of larval arrest in *mutator*-class mutants could be due to the inability of the animals to consume food, absorb nutrients, or due to mis-regulation of factors necessary for proper development of *C. elegans*. Previously, we showed that *mut-16* has a maternal and paternal effect on sterility when animals are raised at elevated temperature (25°C) (Rogers and Phillips, 2020). Thus, the low penetrance larval arrest phenotype of *mut-16* mutants could arise from maternal effects, paternal effects, zygotic effects, or a combination. Further experiments will be needed to determine the underlying cause of larval arrest in *mutator*-class mutants. It is interesting to note that when *mut-16* mutant larvae are synchronized by starvation 28% arrest as larvae, whereas when we previously performed a brood size assay, where no L1 starvation occurred, 15% of *mut-16* mutant larvae arrest (Rogers and Phillips, 2020). This difference suggests that, while the larval arrest phenotype of *mutator* mutants can occur either when larvae hatch from eggs in the presence of food or when starved as L1s, starvation may exacerbate the arrest phenotype. Taken together with the reduced egg laying of *mut-16* mutants (Rogers and Phillips, 2020), these findings suggest that MUT-16, and other *mutator*-class proteins, play key roles in both maintaining fertility and promoting development in *C. elegans*.

## Methods

### *C. elegans* strains.

All animals were grown at 20°C according to standard conditions (Brenner 1974). All strains are in the wild-type (N2) background and have been outcrossed at least four times.

### Larval arrest assay.

Worms were synchronized by bleaching and were then plated on NGM plates, 20 L1 stage animals per plate and one thousand individuals per genotype. After 72 hours at 20°C, the number of individuals that reached adulthood or arrested as L1-L2s were counted.

## Reagents

N2 – wild-type.

NL1810 – *mut-16(pk710) I*.

GR1747 – *mut-15(tm1358) V*.

GR1948 – *mut-14(mg464) smut-1(tm1301) V*.

WM30 – *mut-2(ne298) I*.

NL1820 – *mut-7(pk204) III*.

FX2252 – *rde-8(tm2252) IV*.

USC880 – *nyn-2(tm4844) I; nyn-1(tm5004) IV*.

USC1148 – *mut-2(cmp42[(mut-2::gfp::3xFLAG)]) mut-16(cmp185[mut-16ΔE-K::mCherry::2xHA]) I*.

We used USC1148 (referred to here as *mut-16Δ*) for the *mut-16* deletion allele. It should be noted that USC1148 contains MUT-2::GFP::3xFLAG, which does not affect the function of MUT-2.
